# Sick child’s feeding practices and associated factors among mothers with sick children aged less than 2 years in Gamo zone, southern Ethiopia. Does the participation of fathers contribute to improving nutrition? A facility-based cross-sectional study

**DOI:** 10.3389/fpubh.2023.1256499

**Published:** 2023-10-27

**Authors:** Feven Masresha Hailu, Simegn Wagaye Kefene, Muluken Bekele Sorrie, Misgun Shewangezaw Mekuria, Tamirat Gezahegn Guyo

**Affiliations:** ^1^School of Public Health, College of Medicine and Health Sciences, Arba Minch University, Arba Minch, Ethiopia; ^2^Department of Public Health, Arba Minch College of Health Sciences, Arba Minch, Ethiopia

**Keywords:** sick child, feeding practice, childhood illness, infant and young child feeding, Ethiopia

## Abstract

**Background:**

Children’s nutritional status can decline rapidly during or after a common childhood illness unless additional nutritional requirements associated with the illness are considered. Therefore, this study was aimed at assessing a sick child’s feeding practices and associated factors among mothers who have sick children under 2 years of age in southern Ethiopia.

**Methods:**

A facility-based cross-sectional study design was employed from 1 April 2022 to 30 April 2022. Data were collected through the questionnaire, entered into an open data kit (ODK), and exported into Statistical Packages for Social Sciences (SPSS) version 25.0 for analysis. A systematic random sampling technique was used to select study participants. Bivariable and multivariable logistic regression analyses were used to identify factors associated with sick child feeding practices. An adjusted odds ratio (AOR) with 95% confidence intervals (CIs) was used to determine the strength of the association. Statistical significance was declared at a *p*-value of <0.05.

**Results:**

The overall magnitude of mothers’ good sick child feeding practices was determined to be 45.0% (95% CI: 41.03, 48.97%). Being urban residents, being employed, having antenatal care (ANC) visits, having postnatal care (PNC) visits, counseling about child feeding, and fathers’ involvement in sick child feeding increased the likelihood of sick child feeding practices by 4.4, 2.10, 2.31, 3.54, 2.11, and 2.54 times, respectively.

**Conclusion:**

Sick child feeding practices were associated with having antenatal or postnatal visits, counseling about child feeding, and fathers’ involvement in sick child feeding practices. Therefore, strengthening infant and young child feeding practices by showing special concern for the father’s involvement is important to improve mothers’ sick child feeding practices.

## Introduction

During infancy and early childhood, it is essential to ensure children’s growth, health, and development to their full potential (1). The practice of feeding a sick baby involves increasing fluid intake during the illness, including more frequent breastfeeding and longer feeding sessions both at day and night. The mother or caregiver should also offer the child’s favorite foods for their age group and help and encourage the child to eat (2).

Children’s nutritional status can decline rapidly during or after common childhood illnesses if the additional nutritional requirements associated with the illness are not fulfilled. In such cases, nutrients are diverted from growth and development to build the immune response (3). Improperly fed children have a heightened risk of being affected by both infectious and non-infectious diseases. Since disease consumes calories, food restriction during an illness further results in calorie deprivation; this, combined with recurrent illnesses, can lead to malnutrition (4).

In most developing countries, the proper feeding practices of mothers for their sick children are poor and fair for complementary feed, which ranges from 1 to 69.94%, and poor for breastfeeding children, which ranges from 3 to 17% (5). In sub-Saharan Africa, only 9.3% of children are adequately fed during episodes of diarrhea. There is a variation in subregional coverage; the highest and lowest coverage was recorded in Central Africa and West Africa, which extend between 9.3 and 4.2%, respectively. When examining country-specific coverage, Burkina Faso (0.4%), Rwanda (2.8%), and Nigeria (3.2%) have lower prevalences of good child feeding practices during diarrheal episodes (6). A study conducted in Ethiopia showed that the low prevalence of appropriate child feeding (ACF) practice is associated with the educational status of the mother, place of delivery, birth order of the index child, very close birth spacing, antenatal care (ANC) and postnatal care (PNC) service utilization, socioeconomic status, maternal employment status, access to media, food source of family, inappropriate beliefs, and a lack of knowledge about child feeding practices (5).

Previously conducted studies on sick child feeding practices did not assess the effect of the father’s involvement in child feeding practices or place of residence.

Similarly, to the best of the investigator’s knowledge, no study has been conducted in these study settings. Therefore, this study aimed to assess the feeding practices for sick children and the associated factors among mothers with sick children under 2 years of age in southern Ethiopia.

## Materials and methods

### Study design, period, and settings

A facility-based cross-sectional study was conducted in public health facilities in the Gamo zone, southern Ethiopia. It was conducted from 1 April 2022 to 30 April 2022. Arba Minch is the administrative center of Gamo zone. It is 505 km away from Addis Ababa, the country’s capital city. Based on the database of the Gamo Zone Health Department, the total population of the zone was estimated to be 1,615,510; among the population, 808,114 were men, 807,397 were women, 252,229 were children under five, and 51,537 were infants. There are six public hospitals and 57 health centers in the zone.

### Population

All mothers/caregivers with sick children under 2 years of age attended five outpatient departments (OPD) of the public health facilities in Gamo zone as the source population. All randomly selected mothers/caregivers with sick children aged less than 2 years who attended under five OPDs in selected public health facilities in Gamo zone during the data collection period and who fulfilled the eligibility criteria were included in the study. Children who came to health facilities with people other than their mothers or caregivers were excluded from the study.

### Study variables

#### Dependent variable

Sick child feeding practice (good/poor).

#### Independent variables

Sociodemographic factors include the age of the mothers, occupational status, household wealth index, and educational status of the parents. Maternal and obstetric factors include the place of delivery, parity, birth interval, and knowledge about infant and young child feeding (IYCF) practice. Characteristics of the child include birth order, breastfeeding status of the child, age of the child, and sex of the child. Nutrition-related factors include the food source of the family, minimum dietary diversity, and minimum meal frequency. Health service-related factors include the number of ANC and PNC visits, access to counseling on IYCF, and husband support in child feeding. Environmental health-related factors include the water supply, sanitation, and hygiene-related factors.

### Sample size and sampling procedure

**Figure 1:**
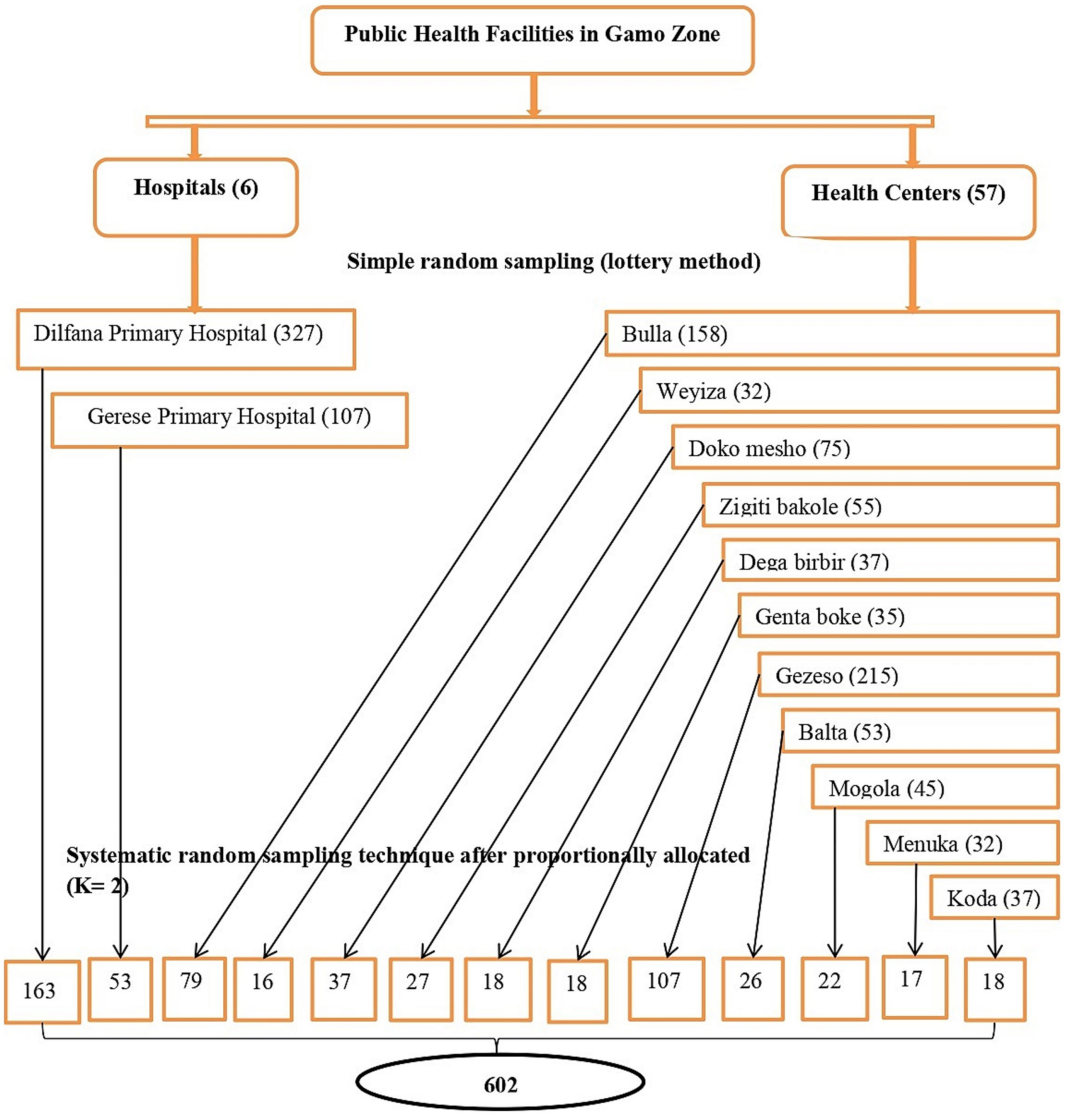
Schematic representation of sampling procedure on sick child feeding practice and associated factors among mothers who have sick children younger than 2 years in public health facilities in Gamo zone, southern Ethiopia.

The sample size was determined using a single population proportion formula using the following assumption: Zα/2 = 1.96, the critical value for normal distribution at 95% confidence intervals, the proportion of sick child feeding practice (P) from previous studies, which was 53.6% (7), the margin of error (d) = 5%, and a 5% non-response rate. It was calculated to be 382, and after considering 1.5 design effects and a 5% non-response rate, the final sample size became 602, which was used to conduct the study. A multi-stage stratified sampling technique was used. First, public health facilities were stratified as health centers and hospitals. Then, 2 hospitals and 11 health centers were selected using simple random sampling (lottery method). Study participants were proportionally allocated to each health facility based on their average monthly client size in under-five OPDs to ensure the sample’s representativeness. Finally, a systematic random sampling technique was used to select study participants. The systematic random sampling technique starts randomly and proceeds with the selection of every $k^{th}$ interval (the determined interval *K* = 2) until the allocated number of study subjects is reached for each health facility (Figure 1).

### Data collection methods and instrument

Data were collected using a pretested, structured interviewer-administered questionnaire with the open data kit (ODK) developed after reviewing different related literature. The questionnaire contains variables related to sociodemographic and economic characteristics, maternal and obstetric factors, health service-related factors, feeding practices of infants and young children, feeding practices of infants and young children during illness, information on sick child feeding, and environmental health-related factors. The questionnaire was initially developed in English and then translated into the Amharic language and back into English to ensure consistency. Thirteen graduated diploma nurses working under five OPDs participated in data collection, and daily-based close supervision was conducted by two supervisors (BSc in Public Health) and the principal investigator during the data collection period.

### Measurements and operational definitions

Household wealth index: It was measured by asking questions about their household assets. The questions were adapted from the Ethiopian Demographic and Health Survey (EDHS) and analyzed using principal component analysis (PCA) (8).

#### Minimum dietary diversity

This was assessed by asking the mothers whether their children had received food from the standard eight food groups on the previous day. The number of food groups the child consumed during the 24 h preceding the survey was used as a proxy for the quality of the diet consumed. It was calculated and divided into two categories: good (consumption of ≥5 food groups) and poor. Dietary diversity (consumption of <5 food groups) is based on the World Health Organization (WHO) guidelines (9).

#### Sick child

Infant or young child with either common childhood illnesses or other illnesses and seeking treatment.

#### Sick child feeding practice

It is defined as a routine for feeding a baby during an illness. Increasing food intake during illness includes more frequent breastfeeding and longer feeds both day and night (2).

Good sick child feeding practice refers to having four or more meals a day for non-breastfed babies aged 6–23 months, taking two or more meals a day (complementary food) for babies aged 6–8 months, and more than three meals a day for babies aged 9–23 months. This condition was not required for those who fed and breastfed at a higher frequency than normal (8 to 12 feeds per day) (10).

#### Poor sick child feeding practices

Mothers/caregivers who gave the usual amount of fluids/foods and those who gave slightly less fluids/foods than usual and the frequency of fluids/foods or interruptions in feeding were considered poor feeding practices for the sick child (10).

#### Meals

The food served and eaten, especially at one of the customary, regular occasions for taking food during the day, such as breakfast, lunch, or supper.

#### Minimum meal frequency

Proportion of breastfed and non-breastfed children aged 6–23 months who received solid, semisolid, or soft foods (but also including milk for non-breastfed children). The minimum frequency was defined as twice for breastfed infants 6–8 months, three times for breastfed children 9–23 months, and four times for non-breastfed children 6–23 months (10).

### Data quality assurance

All data collectors and supervisors were trained adequately for 1 day and performed practical exercises to become familiar with the questionnaire and the software. A reliable tool was used to assess the household wealth index with a Cronbach’s alpha of 0.842 (8). A pretest was conducted before starting the data collection on 5% of the sample to ensure clarity, wording, logical sequence, and skip patterns in the questions. Modifications were made accordingly before starting the actual data collection. In addition, the supervisors and principal investigator supervised the data collection process and checked the filled-out questionnaires every day for completeness and correctness, and necessary additional corrections were made accordingly.

### Statistical analysis

The collected data were downloaded from ODK aggregate as an Excel file and exported to SPSS version 25 for further management and analysis. Descriptive statistics (standard deviation, frequency, and proportions) were computed for all variables according to their type.

The household wealth index was determined using PCA after checking assumptions based on household assets to produce factor scores, which were summed and ranked into five quintiles: “lowest,” “second,” “medium,” “fourth, and “highest,” and households were assigned to each one of the categories. A binary logistic regression was used to identify the association between each independent and dependent variable. In bivariable logistic regression, all variables with a *p*-value of ≤0.25 were selected as candidates and entered into a multivariable logistic regression model to assess the adjusted association between dependent and independent variables. An enter likelihood ratio method was used to fit a multivariable logistic regression model to identify factors remaining in the model. Multi-collinearity was checked using the variance inflation factor (VIF) and tolerance. The maximum observed score VIF value was 1.929, indicating that there was no threat of multi-collinearity. The Hosmer-Lemeshow goodness of fit test was used to check model fitness (*p*-value = 0.412). An adjusted odds ratio (AOR), along with a 95% CI, was used to determine the strength of the association. To declare statistical significance, a *p*-value of <0.05 was used. Finally, the findings were presented using texts, tables, and figures.

## Results

### Sociodemographic and economic characteristics of mothers

A total of 602 mothers voluntarily participated in the study, making a response rate of 100%. All the respondents were the biological mothers of the selected children. The mean (±SD) age of the study participants was 28.86 (±5.720 SD). Among the study participants, 566 (94%) of them were married. The majority of the respondents, 437 (72.6%) were of Gamo ethinicity, and 295 (48.8%) were protestant in religion. More than half of the study participants, that is, 387 (64.3%), were rural dwellers. Regarding the educational status of the study participants, 220 (36.5%) had secondary and above-secondary education levels. Concerning the occupational status of the study participants, 244 (40.5%) were government employees. More than half of the fathers, that is, 384 (63.8%), were not employed by the government. Approximately half of the mothers, i.e., 324 (53.8%), were supported by their husbands during child feeding. Approximately 29.2% of the study participants were poorer. The social status of the remaining participants were the richest (20.1%), poorest (19.9%), middle-class (15.6%), and richer (15.1%), respectively (Table 1).

**Table 1:** Sociodemographic characteristics of mothers who have sick children younger than 2 years attended under five clinics in public health facilities in Gamo zone, southern Ethiopia.

Variables	Frequency	Percent (%)
Age (years)		
18–24	164	27.2
25–35	332	53.5
>35	116	19.3
Residence		
Rural	387	64.36
Urban	215	35.7
Educational status of mothers		
Has no formal education	156	25.9
Primary education	226	37.5
Secondary education	220	36.5
Occupational status of mothers		
Unemployed	358	59.5
Employed	244	40.5
Father support in child feeding		
Yes	324	53.8
No	278	46.2
Household wealth index		
Poorer	176	29.2
Richest	121	20.1
Poorest	120	19.9
Middle	94	15.6
Richer	91	15.1

### Child-related characteristics

Out of the 24-month-old children recorded in this study, more than half of them, that is, 356 (59.1%), were men. The children’s mean (SD) age was 16.25 (5.59 ± SD) months. In terms of birth order, 222 (36.9%) children in this study were the third or higher child in their family. Regarding breastfeeding status, 593 (98.5%) children were breastfed, and only 9 (1.5%) children were not breastfed. Of the breastfed children, the majority of the children, that is, 495 (83.5%), were breastfed less than eight times per day. In addition to the breastfeeding practices, 27 (4.5%) children received pre-lacteal food or fluid. Concerning the colostrum status, 490 (82.6%) children received colostrum. Three hundred fifty-nine (59.6%) children were supplemented with vitamin A. Regarding complementary feeding, the vast majority, 547 (97.3%) of the children, were introduced to complementary feeding after 6 months. Three hundred twelve (51.8%) mothers used bottle-feeding for their children (Table 2).

**Table 2:** Child-related characteristics of mothers who have sick children aged fewer than 2 years who attended under five clinics in public health facilities in Gamo zone, southern Ethiopia.

Variables	Frequency	Percent (%)
Age (in months)		
<6 months	55	9.1
6–12 months	79	13.1
>12	468	77.7
Sex of children		
Male	356	59.1
Female	246	40.9
Breastfeeding status		
Yes	593	98.5
No	9	1.5
Number of breastfeeding		
≥8 times	98	16.5
<8 times	495	83.5
Birth order of the child		
1st child	152	25.2
2nd child	228	37.9
≥3rd child	222	36.9
Vitamin A supplementation		
Yes	359	59.6
No	243	40.4
Age at introducing CF		
≥6 months	547	97.3
<6 months	15	2.7
Bottle feeding		
Yes	312	51.9
No	290	48.2

CF, complementary feeding.

### Maternal and obstetric factors of the mother

More than two-thirds of the mothers of children, that is, 431 (71.6%), gave birth at health institutions and were assisted by health professionals. Three hundred eighty (63.1%) study participants had 1–2 children, and 75 (12.5%) had five or more children.

Regarding birth interval, approximately half of the respondents, i.e., 313 (52.0%), had a birth interval of greater than years between their children, while the remainder had an interval of fewer than 2 years. Approximately half of the respondents, 318 (52.8%), replied that they heard the information on sick child feeding practice, and only 136 (41.2%) respondents heard the information from health professionals during their visit to a health institution.

### Health service-related factors

More than two-thirds of the study participants, i.e., 436 (72.4%), had an ANC visit. Of those who had ANC visits, 401 (92.0%) study participants had ANC visits 1–4 times, and 35 (8.0%) of them had ANC visits greater than four times. Fewer than half, that is, 261 (43.4%), of the study participants had a PNC visit. Of those who had PNC visits, 203 (77.8%) of mothers had PNC visits once, and 58 (22.2%) had PNC visits more than once. More than half, 411 (68.3%), of the mothers interviewed had received counseling on IYCF from health professionals during their visit to a health institution. When their children are sick, approximately half (51.8%) of the mothers took them to the health center (Table 3).

**Table 3:** Health service-related characteristics of mothers who have sick children aged fewer than 2 years who attended under five clinics in public health facilities in Gamo zone, southern Ethiopia.

Variables	Frequency	Percent (%)
ANC visit		
Yes	436	72.4
No	166	27.6
Frequency of ANC visit		
<4 visit	401	92.0
≥4 visit	35	8.0
PNC visit		
Yes	261	43.4
No	341	56.6
Frequency of PNC visit		
1 visit	203	77.8
2 visit	58	22.2
Counseling on IYCF		
Yes	411	68.3
No	191	31.7
Type of health facilities		
Health center	312	51.8
Public hospital	229	38.0
Privet clinics	61	10.1

ANC, antenatal care; IYCF, infant and young child feeding; PNC, postnatal care.

### Nutrition-related factors

The majority of the study participants, 413 (68.6%), purchase their food from the market. The proportion of children with a minimum dietary diversity was 64 (11.7%). The acceptable minimum meal frequency among children was 248 (45.3%). Grains, roots, and tubers were provided to the greatest portion of the children, 518 (97.4%), and only 82 (14.9%) of the selected children were given vitamin A-rich foods.

### Environmental health-related factors

Three hundred forty (56.6%) study participants use private tap water. Five hundred seventy-eight (96.0%) households required less than 20 min’ walk to fetch water from these sources. Five hundred eighteen (71.7%) households used less than 50 L of water per house per day, and 84 (14.0%) households used more than 50 L of water per house per day. With regards to treating drinking water in households, 209 (34.7%) households treated water to make it safe to drink, and more than 341 (56.6%) study participants washed their children daily. Concerning toilet facilities, the majority (99.2%) of participants had latrines, of which 383 (63.6%) households had private latrines and 214 (35.5%) had shared latrines. In terms of waste disposal, 156 (25.9%) households burned their garbage, 330 (54.8%) disposed in a pit, 95 (15.8%) disposed in an open field, and 21 (3.5%) households used a municipal waste disposal system.

### Magnitude of good sick child feeding practice

The overall magnitude of good sick child feeding practice is 271 (45.0%) [95% CI: 41.026, 48.974], which means that 271 mothers provide their children with breast milk or a soft and appetizing complementary diet more frequently during illness than when they are healthy. The proportion of children who are fed more frequently during illness compared to when they are healthy was used to measure sick child feeding practices according to the recommendation.

### Factors associated with sick child feeding practice

In bivariate logistic regression analysis, 12 variables were identified as factors associated with sick child feeding practices for children aged less than 24 months at a *p*-value of <0.25 and were candidates for multivariable logistic regression analysis. The variables were residence, educational status of mothers, occupation status of mothers, birth interval, birth order of child, sex of child, food source of family, place of delivery, counseling about IYCF practice, father involvement in sick child feeding, ANC visit, and PNC visit (Table 4).

**Table 4:** Bivariable and multivariable logistic regression analysis of factors associated with sick child feeding practice among mothers who have sick children aged fewer than 2 years in public health facilities in Gamo zone, southern Ethiopia.

Variables	Sick child feeding practice		COR (95%CI)	AOR (95%CI)
	Good	Poor		
Residence				
Urban	144	71	4.15 (2.91, 5.92)	4.41 (2.74, 7.1)
Rural	127	260	1	1
Educational status				
Secondary and above	122	98	1.57 (1.04, 2.37)	0.56(0.26, 1.20)
Primary education	80	146	0.69 (0.46, 1.05)	0.63 (0.37, 1.07)
no formal education	69	87	1	1
Employment status				
Gov’t employer	152	92	3.32 (2.36, 4.66)	2.08 (1.01, 4.27)
Unemployed	119	239	1	1
Birth interval				
≥2 years	152	161	1.35 (0.98, 1.86)	1.15 (0.73, 1.80)
<2 years	119	170	1	1
Sex of child				
Male	171	185	1.35 (0.97, 1.87)	1.01 (0.67, 1.52)
Female	100	146	1	1
ANC visit				
Yes	225	211	2.78 (1.89, 4.10)	2.31 (1.45, 3.67)^*^
No	46	120	1	1
**PNC visit**				
Yes	151	110	2.53 (1.82, 3.52)	3.54 (2.32, 5.41)^*^
No	120	221	1	1
Father involvement				
Yes	193	131	3.78 (2.68, 5.32)	2.54 (1.66, 3.87)^*^
No	78	200	1	1
Counseling about IYCF				
Yes	215	196	2.64 (1.83, 3.82)	2.11 (1.34, 3.30)^*^
No	56	135	1	1
Food source of family				
Market	210	203	1.51 (0.85, 2.68)	0.65 (0.31, 1.34)
Both	39	96	0.59 (0.31, 1.14)	0.63 (0.29, 1.34)
Home garden	22	32	1	1
Birth order of the child				
1^st^ child	76	76	1.55 (1.02, 2.35)	1.62 (0.92, 2.86)
2^nd^ child	108	120	1.40 (0.96, 2.03)	1.51 (0.95, 2.42)
≥3^rd^ child	87	135	1	1

ANC, antenatal care; AOR, adjusted odds ratio; COR, crude odds ratio; IYCF, infant and young child feeding; PNC, postnatal care.

* Significant at a *p*-value of <0.05.

In multivariable logistic regression analysis, residence, ANC visit, PNC visit, counseling about IYCF practice, father involvement in child feeding, and occupational status of mothers were significantly associated with sick child feeding practices by mothers at a *p*-value of <0.05.

The multivariable analysis indicated that the odds of good sick child feeding practices were 4.4 times higher for mothers living in urban areas than those living in rural areas (AOR = 4.41; 95% CI: 2.74, 7.11). The odds of having a good sick child feeding practice were 2.1 higher for mothers who worked as government employers versus those who were unemployed (AOR = 2.08; 95% CI: 1.01, 4.27). The odds of good sick child feeding practice were 2.3 higher for mothers who received an ANC visit compared to those mothers who did not receive an ANC visit (AOR = 2.310; 95%CI: 1.45, 3.67). Similarly, mothers who received PNC visits were 3.5 times more likely to feed their children frequently during illness compared with their counterparts (AOR = 3.54; 95% CI: 2.32, 5.41). Mothers who had counseling about IYCF were 2.1 times more likely to feed sick children appropriately than those who did not receive counseling about IYCF (AOR = 2.11; 95% CI: 1.34, 3.30). Another significant factor associated with good sick child feeding practice in this study was the father’s involvement during sick child feeding. Mothers of children whose fathers’ support while feeding a sick child were 2.5 times more likely to feed their child than mothers who do not get support from their husband (AOR = 2.54; 95%CI: 1.67, 3.87; Table 4).

## Discussion

This study aimed to assess sick child feeding practices and associated factors among mothers with sick children under 2 years old in Gamo zone public health facilities. According to the findings of this study, the overall magnitude of good sick child feeding practice is 45% (95% CI: 41.03, 48.97%).

The result of this study is consistent with that of a study conducted in Hiwot Fana specialized hospital, Harar, eastern Ethiopia, where 45.0% of the mothers fed their children more frequently when they were sick than when they were healthy (11).

The data gathered from this study are lower than the results of studies conducted in the Mirab Abaya district, 70.7% (12), Burayu town, 53.6% (7), Debre Berhan Town, 70.3% (13), Nellore district, Andhra Pradesh, India, 69.94% (5), the urban slum of Delhi, India, 64% (14), and the tribal district of Maharashtra, 51.5% (15). This difference might be due to most of the studies being conducted in urban areas, as this study was conducted in both urban and rural areas. Access to health services, health-seeking behavior, and cultural differences may cause inconsistencies between findings. Second, this might be due to the high literacy level in urban areas, which can affect the sick child feeding practices by mothers, as better-educated mothers are more likely to understand and apply appropriately the counseling provided by health workers regarding sick infant and young child feeding practices.

However, the finding of this study is higher than the studies conducted in Debre Tabor Hospital, Kenya, Liberia, Malawi, and the rural part of western Maharashtra, India, which found 37.2% (16), 21.1, 16.9, 13.5% (6, 17), and 28.5% (15), respectively. The difference might be due to socioeconomic, cultural, and geographical differences across the study population. Furthermore, variations in societal child-feeding habits and utilization of health services by mothers vary in child feeding practices across countries.

The findings of this study showed that mothers who live in urban areas were more likely to feed their children more frequently during illness than those who lived in rural areas. This could be due to a difference in exposure to information. Hence, the participants who live in urban areas might have better exposure to information concerning good sick child feeding practices than those who live in rural areas. In the current study, government-employee mothers were more likely to continue feeding and giving more frequent feeds than unemployed mothers consistently. This is supported by a study done in a tribal district of Maharashtra (15), India, Burayu town in Ethiopia (7), and a study conducted using EDHS 2016 data in Ethiopia (18). This may be attributed to the difference in the level of education and understanding of government-employee mothers compared to unemployed mothers. Even though government employee mothers spend most of their day out of the home, they strongly adhere to the counseling provided by health providers, look after their babies during illness, and feed them properly.

This study revealed that mothers who had an ANC visit were more likely to feed their sick children than those who had not received an ANC visit. The results of this study are consistent with other studies conducted in Nellore district, Andhra Pradesh, India (5), the Dabat Health and Demographic Surveillance System site in Gondar (19), the rural communities of Sidama (20), Harar in Ethiopia (21), and Debre Birhan in Ethiopia (13). This may be because mothers who follow ANC visit gain nutrition education or counseling about how to feed children during illness from health workers.

The findings of this study showed that mothers who received PNC visits were more likely to feed their children frequently during illness than their counterparts. Almost similar findings were reported in studies in Debre Berhan town (13) and a study conducted using EDHS 2016 data in Ethiopia (18). The reason could be that a lack of PNC visits after delivery may reduce the chance of getting information about how to feed the child properly during illness, and it may result in improper feeding practices of mothers.

The current study providing counseling on IYCF found a significant association with sick child feeding practices. Mothers who got counseling about IYCF were more likely to feed their children during illness appropriately than mothers who did not get counseling about IYCF. These findings corroborate those reported in Burayu town (7), Mirab Abaya district (12), and Arba Minch Zuria district (22). The possible reason might be explained by the potential effect of counseling about IYCF on sick child feeding practice, as mothers who are better informed are more likely to practice proper sick child feeding practices.

In addition, this study revealed that father involvement in sick child feeding was significantly associated with sick child feeding practices. Mothers of children whose fathers supported them when feeding a sick child were more likely to feed their children than their counterparts. This might be because mothers who are helped by their husbands in preparing meals for the baby, feeding the child, and supporting with domestic chores experience reduced workload and gives them encouragement to properly feed the child.

## Limitations

Since the feeding practice of mothers was not scaled, some respondents may have been replying to what they know rather than what they perform.

## Conclusion

In this study, the magnitude of good sick child feeding practices was found to be low in the study settings. Being urban residents, being employed, having antenatal care (ANC) visits, having postnatal care (PNC) visits, counseling about child feeding, and father involvement in sick child feeding were significantly associated with good sick child feeding practices. Therefore, strengthening maternal and child health services such as ANC, PNC, and IYCF, which focus on nutrition counseling, and giving special attention to father involvement in sick child feeding should be the focus in future studies.

## Data availability statement

The original contributions presented in the study are included in the article/supplementary material, further inquiries can be directed to the corresponding author.

## Ethics statement

Ethical approval was obtained from the Institutional Research Ethics Review Board (IRB) of the College of Medicine and Health Sciences, Arba Minch University, Ethiopia with the reference number IRB/1228/2022. Following ethical approval, an official support letter was written by the School of Public Health to the Gamo Zone Health Department. An explanation of the objective of the research was provided to the concerned personnel at the zonal level. Support letters were obtained from the Gamo Zone Health Bureau and submitted to each district and town health office. Similarly, the administrators of each district and town health office wrote a letter to the concerned health facilities. Written informed consent was obtained from the mother or caregiver of each child after providing information about the purpose of the study. The study was conducted in accordance with the Declaration of the Helsinki Convention on Health Research.

## Author contributions

FH: Conceptualization, Data curation, Formal analysis, Funding acquisition, Investigation, Methodology, Project administration, Resources, Software, Validation, Visualization, Writing – original draft, Writing – review & editing. SK: Conceptualization, Formal analysis, Investigation, Methodology, Software, Validation, Visualization, Writing – review & editing. MS: Conceptualization, Methodology, Supervision, Validation, Visualization, Writing – review & editing. MM: Conceptualization, Methodology, Supervision, Validation, Visualization, Writing – review & editing. TG: Investigation, Methodology, Validation, Visualization, Writing – review & editing, Conceptualization.
